# Enhanced axonal regeneration of ALS patient iPSC-derived motor neurons harboring SOD1^A4V^ mutation

**DOI:** 10.1038/s41598-023-31720-7

**Published:** 2023-04-05

**Authors:** Katherine L. Marshall, Labchan Rajbhandari, Arun Venkatesan, Nicholas J. Maragakis, Mohamed H. Farah

**Affiliations:** Neuromuscular Division, Department of Neurology, Johns Hopkins University School of Medicine, The John G. Rangos Sr. Building, Room 239, 855 N. Wolfe Street, Baltimore, MD 21205 USA

**Keywords:** Regeneration and repair in the nervous system, Peripheral nervous system

## Abstract

Amyotrophic lateral sclerosis (ALS) is a devastating neurodegenerative disease, characterized by degeneration of upper and lower motor neurons that leads to muscle weakness, paralysis, and death, but the effects of disease-causing mutations on axonal outgrowth of neurons derived from human induced pluripotent stem cells (iPSC)-derived motor neurons (hiPSC-MN) are poorly understood. The use of hiPSC-MN is a promising tool to develop more relevant models for target identification and drug development in ALS research, but questions remain concerning the effects of distinct disease-causing mutations on axon regeneration. Mutations in *superoxide dismutase 1* (*SOD1*) were the first to be discovered in ALS patients. Here, we investigated the effect of the *SOD1*^A4V^ mutation on axonal regeneration of hiPSC-MNs, utilizing compartmentalized microfluidic devices, which are powerful tools for studying hiPSC-MN distal axons. Surprisingly, SOD1^+/A4V^ hiPSC-MNs regenerated axons more quickly following axotomy than those expressing the native form of SOD1. Though initial axon regrowth was not significantly different following axotomy, enhanced regeneration was apparent at later time points, indicating an increased rate of outgrowth. This regeneration model could be used to identify factors that enhance the rate of human axon regeneration.

## Introduction

Amyotrophic Lateral Sclerosis (ALS) is a devastating disease with a median life expectancy of 24–50 months that is characterized by degeneration of motor neurons^1^. In ALS, spinal motor neurons undergo dying-back axonopathy, where the distal axons of spinal motor neurons retract from their target neuromuscular junctions and degenerate before the death of the neurons themselves^2–4^. Not only is it important to understand pathological process occurring in distal motor axons in ALS, but also the capacity for these axons to regenerate and potentially reinnervate their targets.

Neurons and other brain cell types differentiated from human induced pluripotent stem cells (hiPSCs) have been valuable tools for understanding ALS pathogenesis, identifying new avenues for treatment, and investigating potential therapeutic compounds^5–11^. ALS-causing mutations have been examined in hiPSC-derived spinal motor neurons (hiPSC-MNs), many of which recapitulate crucial aspects of pathology and have been used to identify potential drugs^12,13^. In addition to neuroprotective treatments that increase survival of motor neurons, a potential therapeutic strategy for improving survival and quality of life is promoting motor axon survival and regeneration for the reinnervation of neuromuscular junctions^14^. The capacity of human motor axons to regenerate and reinnervate targets is important not only in the context of ALS, but in the context of peripheral nerve injury.

There is a large unmet clinical need for avenues to stimulate axon regeneration and promote functional recovery. There are no existing treatments for promoting axon regeneration in the peripheral or central nervous systems. Though peripheral nerves regenerate following injury, recovery is often unsuccessful and incomplete due to the slow regeneration rate and long distances to targets^15^.

The *SOD1*A4V is notable for giving rise to a more uniform, early-onset and aggressively progressing disease-course that is lower motor neuron predominant^16–18^. Post-mortem muscle samples from ALS patients, including those with SOD1 mutations, display abnormal NMJ morphology and evidence of reinnervation^19^. Electrophysiological measurements indicate that the loss of motor neurons in SOD1-linked ALS is rapid^20,21^. Despite faster loss of motor units in SOD1-ALS patients, the surviving motor units may sprout and reinnervate NMJs more often than patients with other mutations^22–24^.

However, axon regeneration has not yet been specifically examined in hiPSC-MNs harboring SOD1 mutations. It is difficult to interrogate individual axons of hiPSC-MNs cultured in vitro. They grow in aggregations of neurons that radiate axons and axon fascicles to neighboring aggregations. In order to assess axonal regeneration, we used microfluidic devices to separate neuronal cell bodies from their axons. Microfluidic devices have been used in a variety of contexts where spatial separation of cell bodies and axons is advantageous, including in studying axon regeneration following axotomy^14,25–32^. Therefore, we used microfluidic devices and axotomy to assess axonal regeneration of hiPSC-MNs harboring the SOD1^A4V^ mutation.

## Materials and methods

### Microfluidic device assembly

Microfluidic devices were custom-made using polydimethylsiloxane (PDMS) as previously described^25–27,33^. Sylgard 184 PDMS (Electron Microscopy Sciences, 24236-10) and cross-linker were cast onto silane-coated stainless-steel wafer-molds and hardened in an 80 °C oven. 3 mm-diameter holes for media entry points and axonal compartments were punched into the molded PDMS devices, which were subsequently sonicated and autoclaved. Glass-bottom dishes were sonicated, cleansed with ethanol, and visually inspected for debris. Clean PDMS devices were plasma-treated and bonded to round glass-bottomed dishes (WillCo-dish, HBST-5040), and washed with sterile ddH2O and coated for cell culture. Microfluidic devices contained a central cell body compartment that was adjoined to two, 4 mm x 8 mm axonal compartments with 450 μm long microchannels. Microchannels were 10 μm wide to prevent passage of neuronal cell bodies into the axonal compartments.

### Differentiation of human iPSCs into hiPSC-MNs

**Figure 1:**
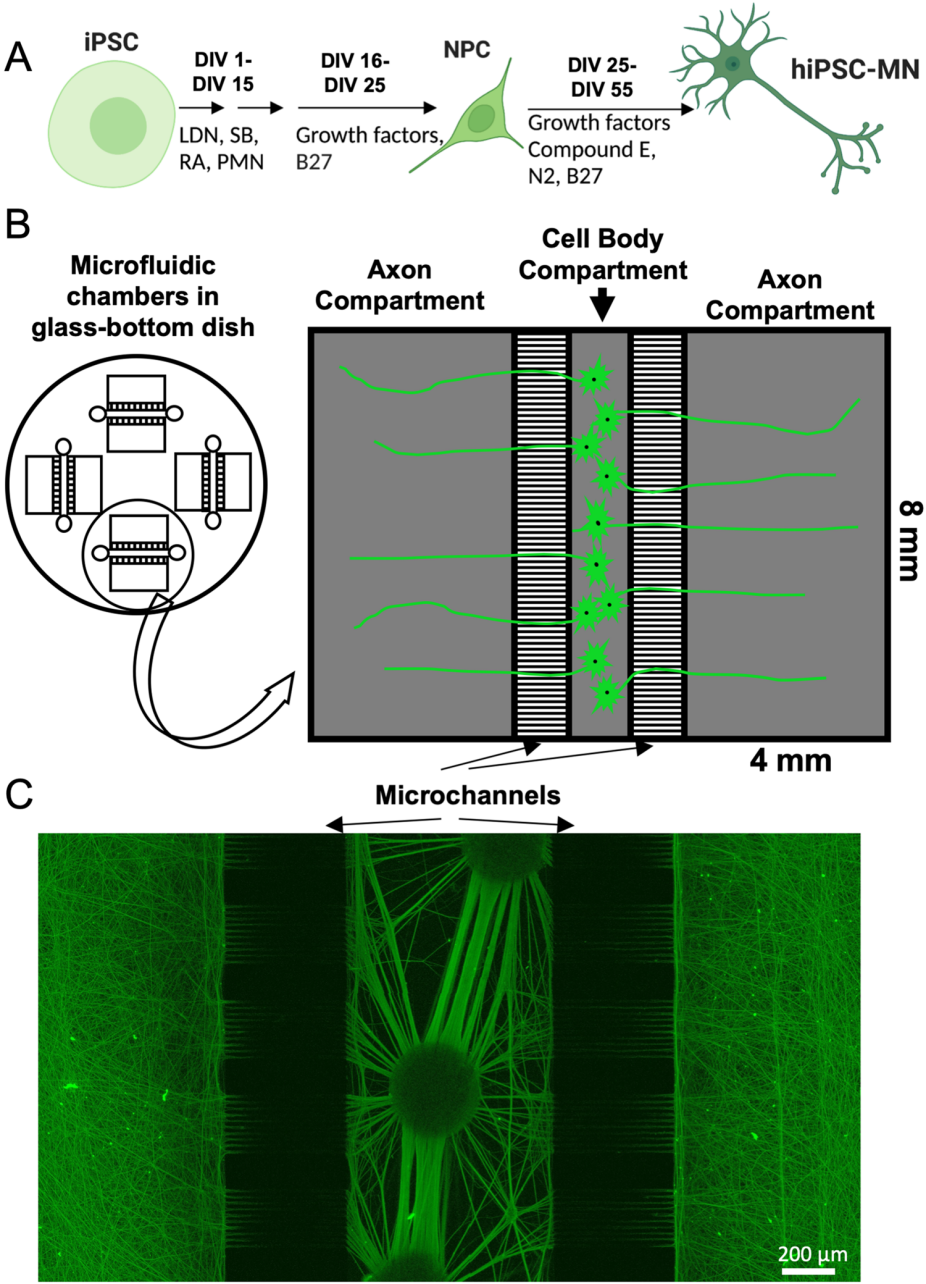
Compartmentalized culture system for investigating axonal outgrowth and regeneration of hiPSC-MNs. (**A**) Schematic representation of iPSC differentiation into spinal hiPSC-MNs. Created with BioRender.com (Supplementary information). (**B**) Compartmentalized microfluidic device for investigating regeneration of healthy control hiPSC-MNs. hiPSC-MN cell bodies are sequestered by microchannels into a central cell body compartment, and axons grow through microchannels into either axonal compartment. (**C**) hiPSC-MNs cultured in microfluidic device stained for smi-31 showing robust outgrowth into both axonal compartments. Scale bar = 200 μm.

**Figure 2:**
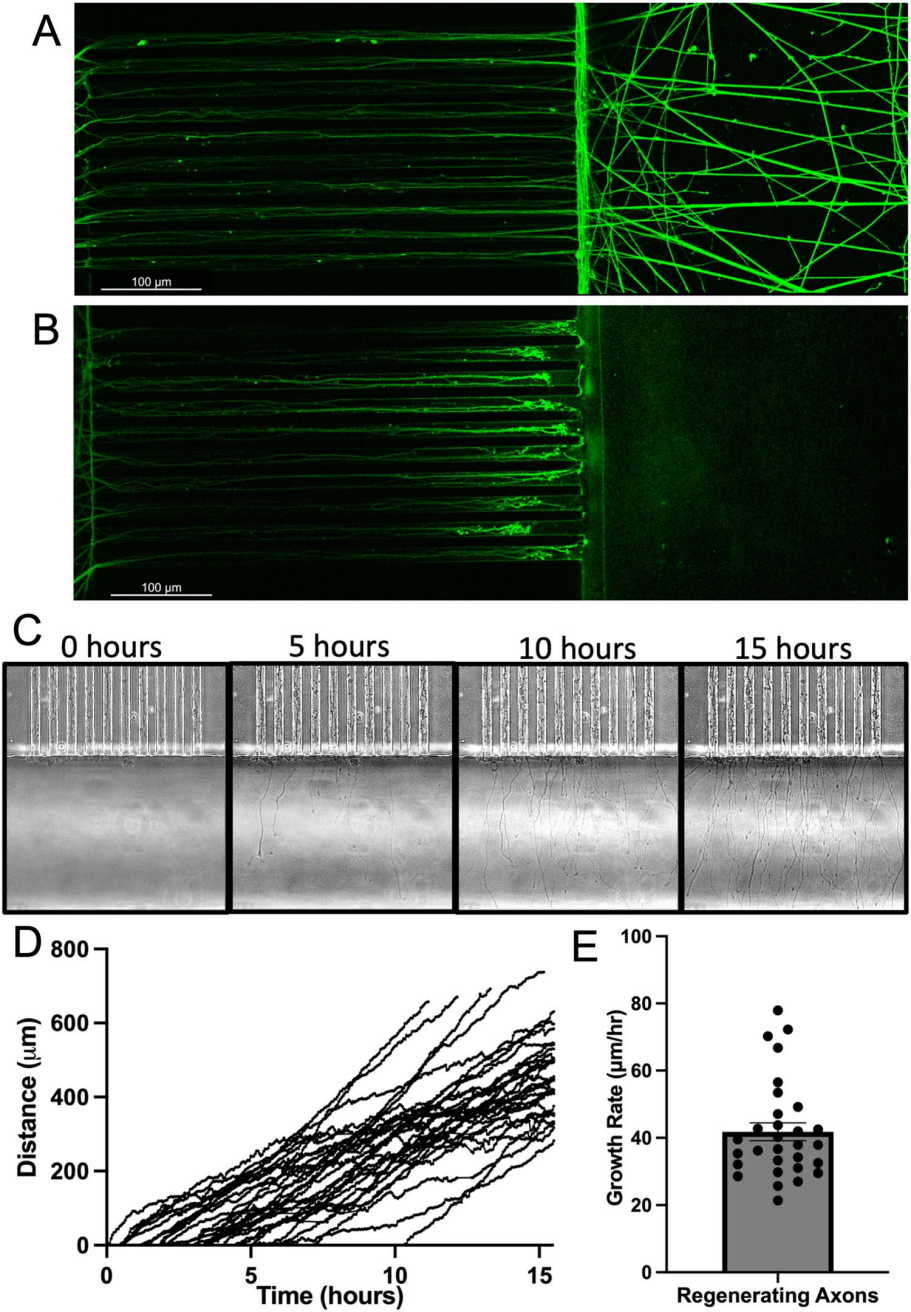
Axotomy and live-imaging of axonal regeneration of control hiPSC-MNs. (**A**,**B**) hiPSC-MN axons stained for smi-31 immediately following axotomy (hour 0). (**A**) Uncut hiPSC-MN axons in microchannels and axonal compartment. (**B**) Axotomized hiPSC-MN axons in microchannels Scale bar = 100 μm. (**C**–**E**) Immediately following axotomy, regenerating axons in the axonal compartment were imaged once every 3 min for 17 h to generate a time-lapse video from which axon terminals were tracked. (**C**) Representative individual frames from one set of microchannels following axotomy at the following fixed time points: 0 h, 5 h, 10 h, 15 h. (**D**) Regeneration distance of individual axons following axotomy. Each line represents an individual axon. n = 30. (**E**) Average regeneration speed. Each point represents the speed of an individual axon. n = 30.

iPSCs were maintained on Matrigel-coated 10 cm petri dishes. A previously described protocol^34,35^, adapted from Roybon et al.^36^ and Boulting et al.^37^, was used to differentiate iPSCs into hiPSC-MNs. The small molecules SB- 431542 (Sigma, S4317) and LDN-193189 (Stemgent, 04–0074), inhibitors of TGF-β and BMP respectively, were added to iPSCs to achieve dual- SMAD inhibition. SOD1^+/+^ and SOD1^+/A4V^ isogenic iPSCs (one mutant and one corrected control line)^9^, were first differentiated into neural progenitor cells (NPCs), which were then frozen into liquid nitrogen between 25 and 30 days in vitro (DIV). Thawed NPCs were differentiated into mature hiPSC-MNs (DIV 55-60). SOD1^+/+^ and SOD1^+/A4V^ isogenic iPSCs were a generous gift from the lab of Kevin Eggan^8,9^. Verification of the mutation and correction was performed via Sanger sequencing^9^. Genotypes of the two lines from three independent platings were verified by extracting DNA from fixed cells within devices following axotomy (Supplementary Fig. 1). Using the protocol in Fig. 1A, neurons differentiated from SOD1^+/+^ and SOD1^+/A4V^ iPSC lines yielded similar percentages (61% for SOD1^+/+^ vs. 60% for SOD1^+/A4V^) of motor neurons as determined by islet 1/2 and β3-tubulin staining (Supplementary Fig. 2). Two healthy control iPSC-lines, CS8PAA and CIPS, were used to validate the assay in Figs. 1 and 2, respectively.

### Culturing hiPSC-MNs in microfluidic devices

NPCs frozen at DIV25-30, were thawed from liquid nitrogen and plated into 10-cm petri dishes coated with 100 μg/mL poly-L-ornithine (PLO, Millipore-Sigma, P3655) and 10 μg/mL laminin (Thermo Fisher, 23017–01). Cells were cultured until DIV40-50, during which time glial progenitor cells were reduced using mitotic inhibitor cytosine arabinoside (Ara-C, 20 μM) hiPSC-MNs (DIV 40-50) were washed with PBS and dissociated with 0.05% trypsin (Millipore-Sigma) for 10 min. Trypsin dissociation was halted with 1:10 trypsin inhibitor (Millipore-Sigma, T6522–1G). hiPSC-MNs were then plated into microfluidic devices coated with PLO (100 μg/mL) and laminin (10 μg/mL). Before neuron plating, PLO/laminin coated devices were washed with PBS (Gibco, 10010-23) and culture media was added to fill all compartments of the devices (1 mL) for at least two hours. Immediately prior to plating, culture media was reduced in axonal compartments and completely removed from the central channel entry ports, while ensuring that media remained in the central channel and no bubbles were introduced. This ensured even distribution of neuronal cell bodies within the central compartment during plating.

To plate neurons into microfluidic devices, cells were concentrated to 50,000 cells/μL of culture medium supplemented with Rho Kinase inhibitor, Y-27632 (Preprotech, 1293823). Concentrated cells (3 μL) were pipetted into the entry port on one side of the central cell-body channel, and 3 μL of media was removed from the opposite side of the cell body channel to draw the neuronal cell bodies into the central compartment. Devices were returned to a 37 °C incubator for two hours to allow hiPSC-MNs to adhere. Culture medium was added to fill the cell body compartment, and axonal compartments were filled with media such that the medium level raised slightly above the upper edges of the axonal compartments. Axons typically grew into the axonal compartment within 2–4 days. To preserve integrity of distal axons, half-volume media changes were performed on axonal compartments. Subsequent experiments with hiPSC-MNs were initiated between DIV 55-60, when neurons had been plated for at least 7 days.

### Axotomy in microfluidic devices

Axotomies were achieved through vigorous vacuum aspiration of the axonal compartment, which severed axons at the junction of microchannel exit and the axonal compartment. Remaining debris was cleared from microchannel exit using a bent pipet tip. This process was repeated 3 times to maximize debris clearance. During and following axotomy, devices were visually inspected under a light microscope to verify microchannel integrity and axon removal. This resulted in complete axotomy, as demonstrated by neurofilament staining immediately post-axotomy.

### Time-lapse imaging and tracking of regenerating axons

Time-lapse images were acquired on the AxioObserver microscope (Zeiss) at the Neuroscience Multiphoton Imaging Core at Johns Hopkins University School of Medicine. For experiments with SOD1 hiPSC-MNs, images of microfluidic devices were acquired between 9.5 and 10 min for 48 h post-axotomy. Three, 5 μm Z-stack images were taken using DIC on the 10 × objective for each image, to account for the large imaging area and inevitable drift over time. Imaging was paused for about 1 h to refocus the microscope between 24 and 48-h time points. To capture regenerating axons, axons within microchannels were used to focus the microscope on the latter portion of the microchannels, exit into the axonal compartment, and initial portion of the axonal compartment. Time-lapse images of cell bodies, and uncut axons were simultaneously acquired to monitor their status following axotomy and verify viability of the uninjured axons. Following time-lapse imaging, the files were stitched together using Zen Blue software (Zeiss), Stitched files were uploaded to Imaris file converter (Imaris v9.2.1; Oxford Instruments). Axons lengths for 24-h and 48-h time points were determined by tracing axons using “filament tracer” on Imaris. Each axonal compartment is separated by 20 clusters of 10 microchannels. To ensure consistent sampling, the 5 longest, individually identifiable regenerating axons from each cluster were traced. Axon speed was determined using the “spots” feature on Imaris. Time-lapse images were inspected and growth cones were tracked manually in each frame. Axon lengths from each genotype across experiments were pooled to generate frequency distributions of axon length, which were then fitted to Gaussian curves for visualization.

### Immunofluorescence staining

Cells were fixed in microfluidic devices using 2% paraformaldehyde (PFA) in PBS, after cell-culture media was diluted until clear using PBS. Cells were permeabilized and blocked by 5% goat serum (Vector Laboratories, S-1000-20)/0.3% Triton X-100 (Sigma-Aldrich 9036-19-5) in 1 X PBS for 1 h at room temperature. Devices were stained with a primary antibody against smi-31, stathmin-2, islet 1/2, and β3-tubulin antibodies at 4 °C overnight (Table 1). Six half-volume washes with PBS-T were performed before the appropriate Alexa Fluor conjugated secondary antibody or phalloidin 647 conjugate was added for 30 min at room temperature. Appropriate concentrations of antibodies, etc., were achieved by added solutions at 2 × concentration to remaining liquid.

**Table 1:** Antibodies/Reagent used for immunofluorescence staining.

1° Antibody/reagent	Target	Company	Dilution
Neurofilament H (SMI-31)	Axonal marker	Biolegend	1:500
β3-tubulin (TUBB3)	Neuronal marker	Biolegend	1:2000
Stathmin-2 (STMN2)	Early regeneration marker	Novus	1:2000
GAP-43	Regeneration Marker	Sigma-Aldrich	1:1000
Phalloidin 647 conjugate	F-actin	Invitrogen	1:400
Islet 1 & 2 (ISL 1/2)	Motor neuron marker	Developmental Studies Hybridoma Bank	1:50

### Statistical analysis

Statistical analysis was performed following consultation with a biostatistician at Johns Hopkins University School of Public Health. Each sector, one of the four on each microfluidic device, was considered one biological replicate. For the 24-h (SOD1^+/+^ n = 13; SOD1^+/A4V^ n = 12) and 48-h (SOD1^+/+^ n = 10; SOD1^+/A4V^ n = 11) time points, three independent neuronal platings were analyzed. For the 5-day time point, two independent neuronal platings were analyzed (SOD1^+/+^ n = 11; SOD1^+/A4V^ n = 9). Sectors were excluded from analysis based on the following criteria: visible damage to microchannels, disruption of the device bond, or imaging drift. Two-tailed student’s t-tests were performed to compare mean regenerated axon length between SOD1^+/A4V^ and SOD1^+/+^ axons. Histograms were generated using cumulative frequencies of axons lengths pooled across all experiments at that time point, and the Kolmogorov–Smirnov test was used to determine statistical significance of population differences.

## Results

### hiPSC-MN cultured in microfluidic devices extend long axons

hiPSC-MNs were differentiated from iPSCs using established protocols (Fig. 1A). In order to interrogate long distal axons of motor neurons, so we sought a microfluidic platform with ample room in the axonal compartment. We used PDMS microfluidic devices with four separate microfluidic sectors on one glass-bottomed dish, each with a central cell body compartment with two axonal compartments on either side (Fig. 1B). The large (8 mm wide × 4 mm long), uncovered axonal compartments would allow mechanical manipulation of axons. The presence of two axonal compartments per sector would also introduce an additional control condition of non-axotomized axons. After being seeded into the central cell body compartment, hiPSC-MNs grew axons into both axonal compartments (Fig. 1C). Axons exiting microchannels and growing into the axonal compartment were visible by light microscopy after 3–4 days (data not shown), and eventually grew long axons (> 1 mm, Fig. 1C). Neurons clustered in the central compartment with comparable axonal growth into both axonal compartments. Therefore, we reasoned that these devices are ideal for studying outgrowth and regeneration of motor axons.

### hiPSC-MNs exhibit robust regeneration of axons

We assessed axon regeneration in microfluidic devices through axotomy at the junction between microchannels and the axonal compartment. We allowed the axons to grow for 7 days after plating the neurons into the microfluidic device. One axonal compartment of each sector of the microfluidic device was axotomized, and the other was left as a control (Fig. 2A,B). Immediately after axotomy, hour 0, uncut axons in the opposite axonal compartment remained intact (Fig. 2A), while the cut side showed complete absence of axons (Fig. 2B), and dying back of axons within the microchannels as visualized by immunofluorescence staining for smi-31.

To explore dynamics of motor axon regeneration, we used live-imaging to monitor regenerating axons for 17 h following axotomy, collecting an image every 3 min. Still frames showed that regenerating axons had emerged from microchannels within 5 hours of axotomy, and the number of regenerating axons had increased by 15 h post axotomy (Fig. 2C). We considered regenerating axon terminals as individual points that we tracked throughout the video in order to determine the speed of individual regenerating axons (Fig. 2D). On average, regenerating axons were traveling at a rate of 41.8 ± 2.6 μm/hour (Fig. 2E), comparable to the rate of axonal outgrowth in humans (~ 1 mm/day)^15^, suggesting that hiPSC-MN outgrowth observed in our model is similar to regeneration observed in vivo.

### SOD1^+/A4V^ mutant axons regenerate farther distances than SOD1^+/+^

Having established a system for evaluating the regeneration of hiPSC-MNs, we compared axonal regeneration of ALS hiPSC-MNs and healthy controls. To test the effects of ALS-linked mutations in SOD1 on hiPSC-MN axonal regeneration, we opted to use a previously characterized hiPSC-MNs generated from an isogenic pair of iPSCs, SOD1^+/A4V^ and SOD1^+/+^^9^. We hypothesized that SOD1^+/+^ corrected hiPSC-MNs would regenerate axons more efficiently than mutant SOD1^+/A4V^ hiPSC-MNs.

**Figure 3:**
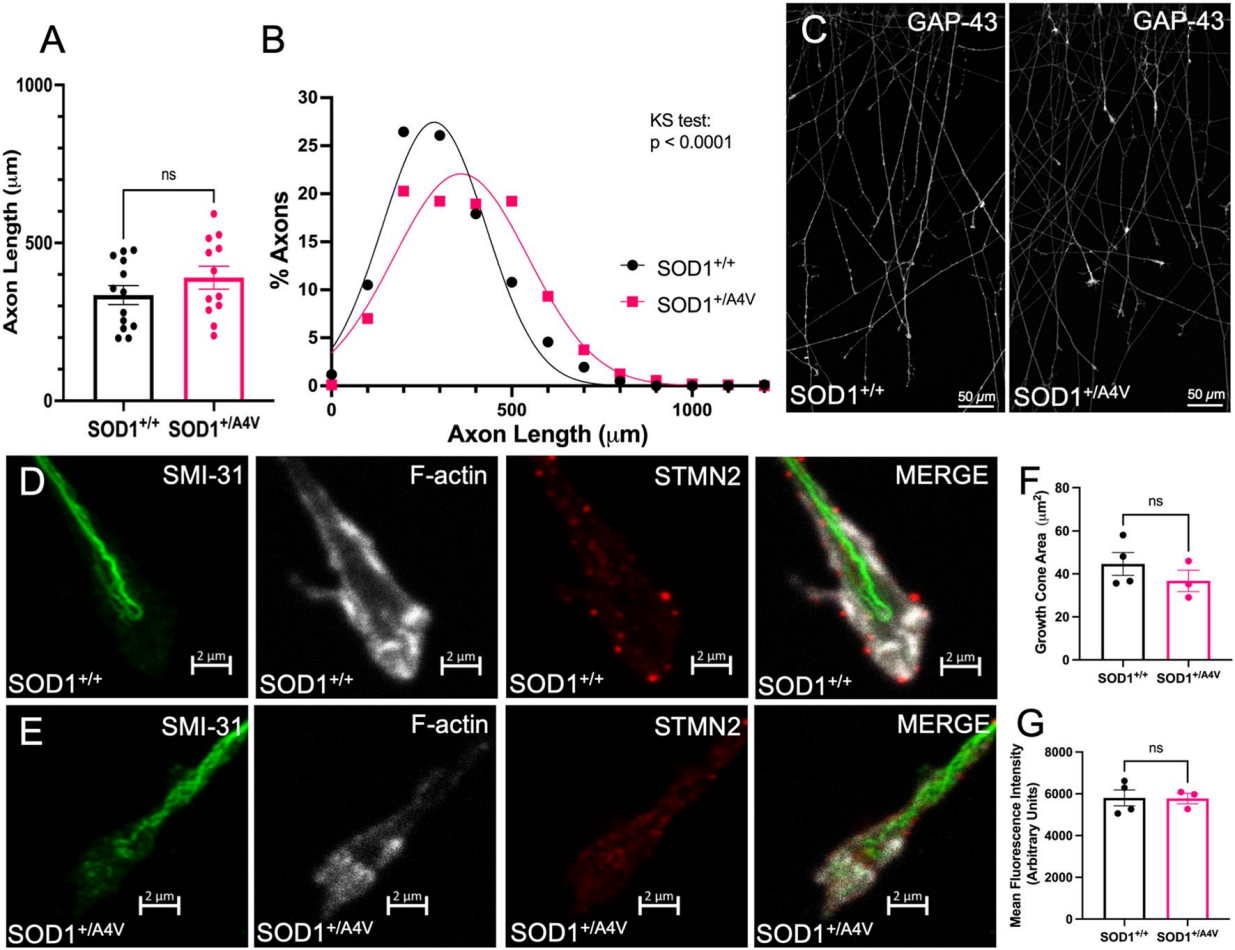
SOD1^+/+^ and SOD1^+/A4V^ axons regenerate similarly 24 h following axotomy. (**A**) Mean axon length 24 h post-axotomy (SOD1^+/+^ n = 13; SOD1^+/A4V^ n = 12). Each data point represents 50–100 traced axons from 1 individual axonal compartment. (**B**) Frequency distribution of axon length 24 h post-axotomy. (**C**) SOD1^+/+^ (left) and SOD1^+/A4V^ (right) regenerating axons 24 h post axotomy stained for GAP-43. Scale bar = 50 $\upmu$m. (**D-E**) Growth cones 24 h post axotomy stained for smi-31 (green), F-actin (white), stathmin-2 (red). Scale bar = 2 $\upmu$m. (**D**) SOD1^+/+^ (top panels) (**E**) SOD1^+/A4V^ (bottom panels). (**F**) Average growth cone area 24 h post-axotomy. (G) Average STMN2 fluorescence intensity in growth cone area 24 h post-axotomy. Bars represent mean ± SEM. ns indicates p > 0.05.

First, we assessed initial axon outgrowth 24 h following plating of hiPSC-MNs into microfluidic devices and observed similar outgrowth between SOD1^+/+^ and SOD1^+/A4V^ axons (39.0 ± 3.7 μm/h and 36.0 ± 9.4 μm/h respectively, Supplementary Fig. 3). The mutant line trended towards a slower initial outgrowth rate than the control. Next, we measured the length of axons 24 h post axotomy, tracing the longest 5 axons from each cluster of microchannels. There was no statistically significant difference in mean length of regenerated axons between SOD1^+/A4V^ and SOD1^+/+^ hiPSC-MNs (Fig. 3A). There was a trend towards longer axons in the mutant hiPSC-MNs; the mean axons length for SOD1^+/A4V^ axons was 390.0 ± 36.3 μm (n = 12), while the SOD1^+/+^ axons had a mean axon length of 334.7 ± 30.0 μm after 24 h (n = 13) (*p* = 0.2493, unpaired t-test). The frequency distribution of axon lengths showed that the distribution of SOD1^+/A4V^ mutant axons was shifted to the right compared to SOD1^+/+^, indicating that a significantly greater proportion of SOD1^+/A4V^ mutant axons were regenerating longer axons (Kolmogorov–Smirnov test *p* < 0.0001). For SOD1^+/A4V^ axons, 19.2% had regenerated 500–600 μm in 24 h, as opposed to 10.8% for SOD1^+/+^ axons (Fig. 3B), reflecting a trend towards longer regenerated axons in SOD1^+/A4V^ in hiPSC-MNs. GAP-43, a regeneration associated gene, was present in regenerating axons from both lines 24 h post axotomy with no overt differences (Fig. 3C). We examined growth cones of regenerating axons 24 h post axotomy, and did not observe any difference in growth cone area as identified by F-actin staining (Fig. 3D–F). We additionally examined stathmin-2, which has been implicated in ALS-related regeneration deficits, in growth cones of regenerating axons and observed no difference in average stathmin-2 fluorescence intensity (Fig. 3D,E,G).

### SOD1^+/A4V^ mutant neurons regenerate longer axons than SOD1^+/+^ two days and five days post axotomy

**Figure 4:**
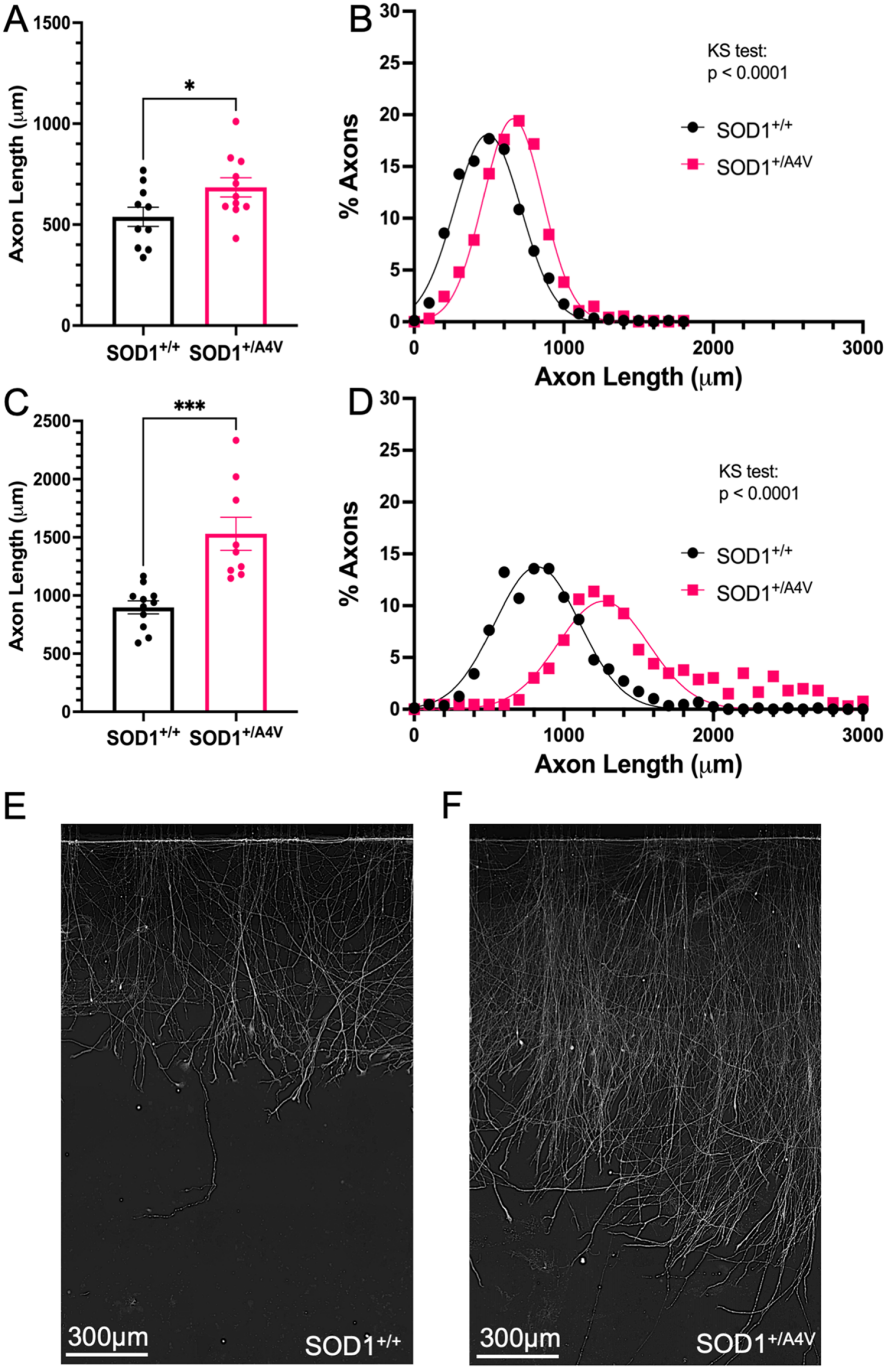
Enhanced regeneration of SOD1^+/A4V^ mutant axons. (**A**) Mean axon length 48 h post-axotomy (SOD1^+/+^ n = 10; SOD1^+/A4V^ n = 11). *indicates p = 0.0438. (**B**) Frequency distribution of axon length. (**C**) Axon length 5 days post-axotomy. (**D**) Mean axon length 5 days post-axotomy (SOD1^+/+^ n = 11; SOD1^+/A4V^ n = 9). *** indicates *p* = .0003. (**E**) Frequency distribution of axon length 24 h. Each data point on bar graphs (**A**,**C**) represents 50–100 traced axons from 1 individual axonal compartment. Bars represent mean ± SEM. (**D**–**E**) Representative images of regenerating axons 5 days post axotomy SOD1^+/+^ (**D**) SOD1^+/A4V^ (**E**).

48 h post-axotomy, the mean length of SOD^+/A4V^ regenerating axons was greater than SOD1^+/+^ hiPSC-MNs (Fig. 4A). The mean axon length for regenerating SOD1^+/A4V^ axons was 684.2 ± 47.7 μm (n = 11), while the mean length of regenerated axons for SOD1^+/+^ was 538.2 ± 47.7 μm (n = 10) (*p* = 0.034, unpaired t-test). Longer mutant axons were also reflected in the frequency distribution (Fig. 4B), where the distribution of SOD^+/A4V^ mutant axons was shifted even farther to the right compared to SOD1^+/+^ at the 48-h time point (Kolmogorov–Smirnov *p* < 0.0001). For SOD1^+/A4V^ axons, 85% of traced axons were greater than 500 μm as opposed to 60% for SOD1^+/+^ axons. Five days post-axotomy, the mean length of SOD^+/A4V^ axons was greater than SOD1^+/+^ hiPSC-MNs (Fig. 4C). The mean axon length for SOD1^+/A4V^ regenerated axons was 1530.0 ± 142.0 μm (n = 9), while the mean length for SOD1^+/+^ axons was 898.1 ± 55.59 μm (n = 11) (***p = 0.0003, unpaired t-test). Longer mutant axons were also visible in the frequency distribution, where the distribution of SOD^+/A4V^ mutant axons was even farther shifted to the right compared to SOD1^+/+^ at the 5-day time point time point (Fig. 4D, Kolmogorov–Smirnov *p* < 0.0001). For SOD^+/A4V^ axons, over 75% of traced axons were greater than 1200 μm, while over 75% percent of SOD^+/+^ axons were shorter. This is apparent in SMI-31 staining 5 days post-axotomy, in which hiPSC-MNs regenerated longer axons from SOD^+/A4V^ (Fig. 4F) compared to SOD^+/+^ (Fig. 4E).

## Discussion

We developed a system for studying motor axon outgrowth and regeneration of hiPSC-MNs. Distal axonopathy of motor neurons is an early pathological hallmark of ALS, thus investigating motor axon regeneration using hiPSC-derived neurons is highly relevant. Segregating axons from cell bodies makes biological sense in that neuronal cell bodies are often spatially separated from their targets in vivo. This is true of spinal motor neurons with their cell bodies in the spinal cord, and their distal axons project onto muscle that can be a meter away. We examined axonal regeneration by culturing hiPSC-derived neurons in microfluidic devices with large axonal compartments, which allowed us to observe axon length. Considering that the variation in genetic background in human patients is a potential confounding factor^38^, we examined an isogenic pair of hiPSC-MNs: SOD1^+/A4V^ and SOD1^+/+^. While initial regrowth of the mutant SOD1^+/A4V^ axons was not significantly different from SOD1^+/+^ isogenically-corrected axons, differences in regeneration were apparent 2–5 days post-axotomy.

In previous studies, a number of perturbations were found in the SOD1^+/A4V^ hiPSC-MNs compared to the isogenic control. Cell survival was reduced in SOD1^+/A4V^ hiPSC-MNs, in addition to differences in morphology, including smaller somas and shorter processes^9,39^. Characterization of these cells through a combination of RNA sequencing and functional investigation revealed many significant transcriptional changes, including increased oxidative stress, increased ER stress, unfolded protein response, altered subcellular transport, and impaired mitochondrial transport and structure^9,40^. Electrophysiological characterization of SOD1^+/A4V^ and SOD1^+/+^ demonstrated that mutant hiPSC-MNs were hyperexcitable and had reduced delayed-rectifier potassium current amplitudes^8,41^.

Additionally, neurite outgrowth defects have been observed in hiPSC-MNs with a variety of SOD1 mutations. Genetic correction of these mutations resulted in the amelioration of observed mutant phenotypes. hiPSC-MNs with the *SOD1*D90A mutation, the most common SOD1 mutation in ALS patients outside of North America, demonstrate aberrant regulation of neurofilaments^42^. Misfolded SOD1 was present in axons of both SOD1^+/A4V^ and SOD1^+/G93A^ hiPSC-MNs^39^. hiPSC-MNs harboring the *SOD1*A4V, *SOD1*G93A, and *SOD1*E100G mutations projected fewer processes with shorter initial axon outgrowth compared to their respective isogenic controls^9,39,43^. However, these studies examined neurite outgrowth without spatial separation of neuronal cell bodies and axons, and did not specifically examine regenerating axons following axotomy. In SOD1^+/A4V^ and SOD1^+/G93A^ hiPSC-MN, only relatively short axons were examined (50–250 μm)^9,39^. Considering this previous characterization of *SOD1* mutant hiPSC-MNs, in addition to the particularly aggressive, lower motor neuron predominant disease course that *SOD1*^*A4V*^ mutation exhibits in patients^16^, we previously hypothesized that axonal regeneration would be impaired in SOD1^+/A4V^ mutant axons.

We examined initial regrowth following axotomy, but also followed regenerating axons to later time points to better observe regeneration rate. During initial regrowth, at 24 h post axotomy, the average axon length was not significantly different, and there was a trend towards longer SOD1^+/A4V^ axons. At this early time point, we examined other methods of characterizing outgrowth, by looking at growth cone size and regeneration associated genes. GAP-43 was expressed similarly in regenerating axons from both SOD1^+/+^ SOD1^+/A4V^ hiPSC-MNs. We did not observe any statistically significant differences in growth cone size in SOD1^+/A4V^ hiPSC-MNs, as had been observed in adult motor neurons from SOD1^G93A^ mice^44^. We also examined stathmin-2 expression within the growth cone area, due to it being implicated in hiPSC-MN regeneration^28,29,45^. RNA sequencing data of SOD1^+/A4V^ motor neurons revealed downregulation of stathmin-2 transcripts (fold change 0.459, log2 fold change − 1.124)^9^, but we detected no significant differences in growth cone stathmin-2 fluorescence intensity between SOD1^+/A4V^ and SOD1^+/+^ 24 h following axotomy.

48 h post-axotomy, and 5 days post-axotomy, enhanced regeneration of SOD1^+/A4V^ axons was apparent in mean length of regenerated axons and in the frequency distribution of axon length. Enhanced regeneration of SOD1^+/A4V^ axons was an unexpected result, as the bulk of the literature has suggested process outgrowth in hiPSC-MNs is negatively impacted by harboring mutations in *SOD1.* Though neurite outgrowth of SOD1^+/A4V^ was shorter in initial characterizations, only the initial outgrowth of shorter axons was examined^9^. Hastened regeneration following axotomy opened that possibility *SOD1* mutant may cause the increased expression of regeneration-associated genes. Consistent with this idea, RNA sequencing of SOD1^+/+^ and SOD1^+/A4V^ neurons revealed upregulation of transcripts related to the cytoskeleton, microtubules, microtubule-based processes and movement, and motor proteins^9^. It is possible that mutant SOD1 upregulation of these and/or other transcripts may underlie the increased rate of axonal regeneration.

In SOD1^G93A^ ALS rodent neurons, both enhanced and impaired axonal regeneration has been observed. Enhanced regeneration was observed in SOD1^G93A^ mice following sciatic nerve crush injury^46^ and in SOD1 mutant motor neurons isolated at presymptomatic stages^44^. However, another study found iPSC-derived motor neurons from SOD1^G93A^ mice to have reduced neurite length compared to WT^47^. Multiple studies have demonstrated SOD1^G93A^ mice and rats to have impaired axonal regeneration after sciatic and facial nerve crush injuries^48–51^.

More relevant to our study, conflicting results in regards to axon regeneration have also been reported with hiPSC-MNs. In two recent studies that compared regeneration of FUS^P525L^ hiPSC-MNs to an isogenic control, one group found that regeneration was enhanced following axotomy^31^, while another observed impaired regeneration^32^. There are several factors that may account for this disparity, including the age and maturity of hiPSC-MNs in culture, axotomy method, and the length of neuronal processes examined. Notably, in the study that noted impaired regeneration, shorter neuronal processes were examined (microchannel length: 75–150 μm) following combined mechanical/chemical axotomy^32^, while enhanced regeneration was observed in longer axons (microchannel length: 500 μm) following axotomy by three methods: trypsin, accutase, mechanical^31^. This suggests that the length of neuronal processes examined may be important for determining the regenerative capacity of hiPSC-MNs. Axon length is particularly important in the context of ALS, as longer axons are more vulnerable to degeneration in vivo^52^. Additionally, processes that reach the axonal compartment through microchannels shorter than 450 μm may include neuronal processes other than axons^30^. We observed an enhanced regeneration phenotype in SOD1^+/A4V^ hiPSC-MNs following mechanical axotomy in 450 μm microchannels in a similar manner to the study demonstrating enhanced regeneration of FUS^P525L^ hiPSC-MNs^31^.

Another potentially relevant aspect of ALS pathology is TDP-43 proteinopathy, where TDP-43 tends to be excluded from the nucleus and present in cytoplasmic aggregates, which is present in the majority of ALS cases, but not in *SOD1* and *FUS*^53^. Though axonal regeneration has not specifically been examined in hiPSC-MNs with mutations in TDP-43, hiPSC-MNs in which TDP-43 had been knocked down demonstrated striking deficits in regeneration following axotomy^28,29^. Impaired regeneration was rescued by reintroduction of stathmin-2, which is essential for axonal regeneration^28,29,54^. It is worth considering that different ALS mutations may not be consistent in their effects on axon regeneration capacity, and that treatments that improve axon outgrowth and regeneration may not be broadly generalizable for all ALS. Ropinirole effectively ameliorated multiple phenotypes, including neurite outgrowth defects, in hiPSC-MNs from sporadic and familial ALS patients, with the exception of *SOD1* patient hiPSC-MNs in which phenotypes were not reversed^12,55^.

Though counterintuitive, enhanced regeneration observed in mutant hiPSC-MNs may represent an opportunity to learn about factors that impact regeneration rate in human cells. Studying axon regeneration using microfluidic devices provides a method to identify and study factors that regulate rate of axon outgrowth and regeneration in human cells. Gaining an understanding of the factors that enhance motor axon regeneration is important in the context of ALS, but also in nerve injury. In human nerve injury, the distance to reinnervate a target is often several centimeters to a meter from the site of axonal retraction. Reducing the latency period, as done by genetic overexpression of regeneration-associated genes or upregulated by conditional lesion paradigms in rodents^13,56–58^, would yield limited benefit compared to increasing the rate of axonal outgrowth in the human scale^59–61^. Using readily available microfluidic devices and tools, assays for potential compounds that increase axon regeneration rate over extended periods of time are feasible. Combination of live-imaging of regenerating axons with more powerful analysis methods, such as automated tracking and quantification, may lead to higher-throughput screens for factors that promote hiPSC-MN axonal regeneration^55^. Furthermore, axonal isolation through compartmentalized culture of neurons allows for axon-specific proteomic and transcriptomic analyses^62^, that may in the future reveal yet unknown factors involved in the rate of axon outgrowth and regeneration.

## Supplementary Information

Supplementary Figures.

Supplementary Information.

## Data Availability

The datasets generated during the current study are available from the corresponding author on reasonable request.

## References

[1] Longinetti E, Fang F (2019). Epidemiology of amyotrophic lateral sclerosis: An update of recent literature. Curr. Opin. Neurol..

[2] Fischer LR, Glass JD (2007). Axonal degeneration in motor neuron disease. Neurodegener. Dis..

[3] Fischer LR (2004). Amyotrophic lateral sclerosis is a distal axonopathy: Evidence in mice and man. Exp. Neurol..

[4] Brown RH, Al-Chalabi A (2017). Amyotrophic lateral sclerosis. N. Engl. J. Med..

[5] Richard JP, Maragakis NJ (2015). Induced pluripotent stem cells from ALS patients for disease modeling. Brain Res..

[6] Hawrot J, Imhof S, Wainger BJ (2020). Modeling cell-autonomous motor neuron phenotypes in ALS using iPSCs. Neurobiol. Dis..

[7] Lee S, Huang EJ (2017). Modeling ALS and FTD with iPSC-derived neurons. Brain Res..

[8] Wainger BJ (2014). Intrinsic membrane hyperexcitability of amyotrophic lateral sclerosis patient-derived motor neurons. Cell Rep..

[9] Kiskinis E (2014). Pathways disrupted in human ALS motor neurons identified through genetic correction of mutant SOD1. Cell Stem Cell.

[10] Egawa N (2012). Drug screening for ALS using patient-specific induced pluripotent stem cells. Sci. Transl. Med..

[11] Johns AE, Maragakis NJ (2022). Exploring motor neuron diseases using iPSC platforms. Stem Cells.

[12] Okano H, Yasuda D, Fujimori K, Morimoto S, Takahashi S (2020). Ropinirole, a new ALS drug candidate developed using iPSCs. Trends Pharmacol. Sci..

[13] Wainger BJ (2021). Effect of ezogabine on cortical and spinal motor neuron excitability in amyotrophic lateral sclerosis: A randomized clinical trial. JAMA Neurol..

[14] Marshall KL, Farah MH (2021). Axonal regeneration and sprouting as a potential therapeutic target for nervous system disorders. Neural Regen. Res..

[15] Grinsell D, Keating CP (2014). Peripheral nerve reconstruction after injury: A review of clinical and experimental therapies. Biomed. Res. Int..

[16] Rosen DR (1994). A frequent ala 4 to val superoxide dismutase-1 mutation is associated with a rapidly progressive familial amyotrophic lateral sclerosis. Hum. Mol. Genet..

[17] Saeed M (2009). Age and founder effect of S0D1 A4V mutation causing ALS. Neurology.

[18] Juneja T, Pericak-Vance MA, Laing NG, Dave S, Siddique T (1997). Prognosis in familial amyotrophic lateral sclerosis: Progression and survival in patients with glu100gly and ala4val mutations in Cu, Zn superoxide dismutase. Neurology.

[19] Bruneteau G (2015). Endplate denervation correlates with Nogo-A muscle expression in amyotrophic lateral sclerosis patients. Ann. Clin. Transl. Neurol..

[20] Aggarwal A, Nicholson G (2002). Detection of preclinical motor neurone loss in SOD1 mutation carriers using motor unit number estimation. J. Neurol. Neurosurg. Psychiatry.

[21] Aggarwal A, Nicholson G (2001). Normal complement of motor units in asymptomatic familial (SOD1 mutation) amyotrophic lateral sclerosis carriers. J. Neurol. Neurosurg. Psychiatry.

[22] Bocci T (2011). Differential motor neuron impairment and axonal regeneration in sporadic and familiar amyotrophic Lateral Sclerosis with SOD-1 mutations: Lessons from neurophysiology. Int. J. Mol. Sci..

[23] Benatar M, Wuu J (2012). Presymptomatic studies in ALS: Rationale, challenges, and approach. Neurology.

[24] Jokela M (2016). Distinct muscle biopsy findings in genetically defined adult-onset motor neuron disorders. PLoS ONE.

[25] Rajbhandari L (2014). Toll-like receptor 4 deficiency impairs microglial phagocytosis of degenerating axons. Glia.

[26] Kurapati S (2017). Role of the JNK pathway in varicella-zoster virus lytic infection and reactivation. J. Virol...

[27] Hosmane S, Yang IH, Ruffin A, Thakor N, Venkatesan A (2010). Circular compartmentalized microfluidic platform: Study of axon–glia interactions. Lab Chip.

[28] Melamed Z (2019). Premature polyadenylation-mediated loss of stathmin-2 is a hallmark of TDP-43-dependent neurodegeneration. Nat. Neurosci..

[29] Klim JR (2019). ALS-implicated protein TDP-43 sustains levels of STMN2, a mediator of motor neuron growth and repair. Nat. Neurosci..

[30] Taylor AM (2005). A microfluidic culture platform for CNS axonal injury, regeneration and transport. Nat. Methods.

[31] Garone MG (2021). ALS-related FUS mutations alter axon growth in motoneurons and affect HuD/ELAVL4 and FMRP activity. Commun. Biol..

[32] Stoklund Dittlau K (2021). Human motor units in microfluidic devices are impaired by FUS mutations and improved by HDAC6 inhibition. Stem Cell Rep..

[33] Sadaoka T (2016). In vitro system using human neurons demonstrates that varicella-zoster vaccine virus is impaired for reactivation, but not latency. Proc. Natl. Acad. Sci..

[34] Taga A (2021). Establishment of an electrophysiological platform for modeling ALS with regionally-specific human pluripotent stem cell-derived astrocytes and neurons. J. Vis. Exp..

[35] Taga A (2019). Role of human-induced pluripotent stem cell-derived spinal cord astrocytes in the functional maturation of motor neurons in a multielectrode array system. Stem Cells Transl. Med..

[36] Boulting GL (2011). A functionally characterized test set of human induced pluripotent stem cells. Nat. Biotechnol..

[37] Roybon L (2013). Human stem cell-derived spinal cord astrocytes with defined mature or reactive phenotypes. Cell Rep..

[38] Vasques JF, Mendez-Otero R, Gubert F (2020). Modeling ALS using iPSCs: Is it possible to reproduce the phenotypic variations observed in patients in vitro?. Regen. Med..

[39] Kim BW, Ryu J, Jeong YE, Kim J, Martin LJ (2020). Human motor neurons with SOD1-G93A mutation generated from CRISPR/Cas9 gene-edited iPSCs develop pathological features of amyotrophic lateral sclerosis. Front. Cell. Neurosci..

[40] Shlevkov E (2019). A high-content screen identifies TPP1 and aurora B as regulators of axonal mitochondrial transport. Cell Rep..

[41] Kiskinis E (2018). All-optical electrophysiology for high-throughput functional characterization of a human iPSC-derived motor neuron model of ALS. Stem Cell Rep..

[42] Chen H (2014). Modeling ALS with iPSCs reveals that mutant SOD1 misregulates neurofilament balance in motor neurons. Cell Stem Cell.

[43] Bhinge A, Namboori SC, Zhang X, VanDongen AMJ, Stanton LW (2017). Genetic correction of SOD1 mutant iPSCs reveals ERK and JNK activated AP1 as a driver of neurodegeneration in amyotrophic lateral sclerosis. Stem Cell Rep..

[44] Osking Z (2019). ALS-linked SOD1 mutants enhance neurite outgrowth and branching in adult motor neurons. iScience.

[45] Grenningloh G, Soehrman S, Bondallaz P, Ruchti E, Cadas H (2004). Role of the microtubule destabilizing proteins SCG10 and stathmin in neuronal growth. J. Neurobiol..

[46] Sharp PS, Tyreman N, Jones KE, Gordon T (2018). Neurobiol. Dis..

[47] Park JH, Park HS, Hong S, Kang S (2016). Motor neurons derived from ALS-related mouse iPS cells recapitulate pathological features of ALS. Exp. Mol. Med..

[48] Deng B (2018). Progressive degeneration and inhibition of peripheral nerve regeneration in the SOD1-G93A mouse model of amyotrophic lateral sclerosis. Cell. Physiol. Biochem..

[49] Mesnard NA, Haulcomb MM, Tanzer L, Sanders VM, Jones KJ (2013). Delayed functional recovery in presymptomatic mSOD1G93A mice following facial nerve crush axotomy. J. Neurodegener. Regen..

[50] Schram S (2019). Mutant SOD1 prevents normal functional recovery through enhanced glial activation and loss of motor neuron innervation after peripheral nerve injury. Neurobiol. Dis..

[51] Joshi AR, Muke I, Bobylev I, Lehmann HC (2019). ROCK inhibition improves axonal regeneration in a preclinical model of amyotrophic lateral sclerosis. J. Comp. Neurol..

[52] Tallon C, Russell KA, Sakhalkar S, Andrapallayal N, Farah MH (2016). Length-dependent axo-terminal degeneration at the neuromuscular synapses of type II muscle in SOD1 mice. Neuroscience.

[53] Prasad A, Bharathi V, Sivalingam V, Girdhar A, Patel BK (2019). Molecular mechanisms of TDP-43 misfolding and pathology in amyotrophic lateral sclerosis. Front. Mol. Neurosci..

[54] Chauvin S, Sobel A (2015). Neuronal stathmins: A family of phosphoproteins cooperating for neuronal development, plasticity and regeneration. Prog. Neurobiol..

[55] Fujimori K (2018). Modeling sporadic ALS in iPSC-derived motor neurons identifies a potential therapeutic agent. Nat. Med..

[56] Seijffers R, Mills CD, Woolf CJ (2007). ATF3 increases the intrinsic growth state of DRG neurons to enhance peripheral nerve regeneration. J. Neurosci..

[57] Ma CHE (2011). Accelerating axonal growth promotes motor recovery after peripheral nerve injury in mice. J. Clin. Investig..

[58] Costigan M (2002). Replicate high-density rat genome oligonucleotide microarrays reveal hundreds of regulated genes in the dorsal root ganglion after peripheral nerve injury. BMC Neurosci..

[59] Griffin JW, Pan B, Polley MA, Hoffman PN, Farah MH (2010). Measuring nerve regeneration in the mouse. Exp. Neurol..

[60] Sulaiman WA, Kline DG (2006). Nerve surgery: A review and insights about its future. Clin. Neurosurg..

[61] Gordon T (2009). Accelerating axon growth to overcome limitations in functional recovery after peripheral nerve injury. Neurosurgery.

[62] Nijssen J, Aguila J, Hoogstraaten R, Kee N, Hedlund E (2018). Axon-seq decodes the motor axon transcriptome and its modulation in response to ALS. Stem Cell Rep..

