# Supplementary material for: Enhanced axonal regeneration of ALS patient iPSC-derived motor neurons harboring SOD1A4V mutation
Source: Sci Rep. 2023 Apr 5;13:5597. doi: 10.1038/s41598-023-31720-7 (PMC10076424; doi:10.1038/s41598-023-31720-7)
Supplement: Supplementary file 1 — Supplementary Figures. [file 41598_2023_31720_MOESM1_ESM.pdf]

ENHANCED AXONAL REGENERATION OF ALS PATIENT IPSC-DERIVED MOTOR NEURONS HARBORING SOD1^{A4V} MUTATION

Authors: Katherine L. Marshall, Labchan Rajbhandari, Arun Venkatesan, Nicholas J. Maragakis, Mohamed H. Farah

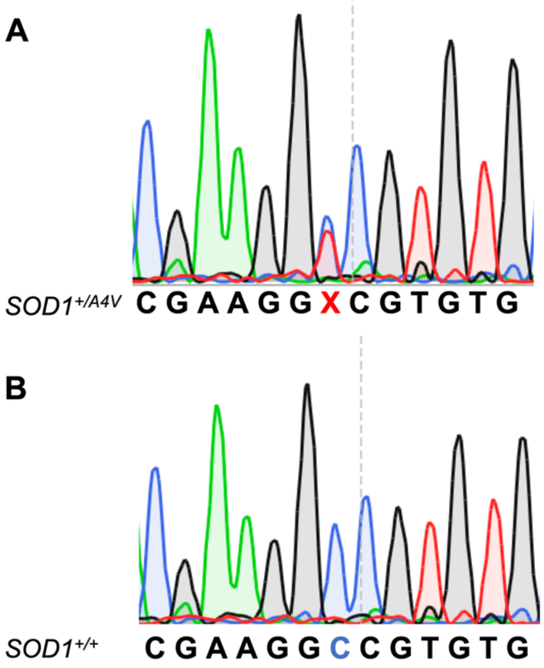

Supplementary figure 1: Verification of SOD1$^{+/+}$ and SOD1$^{+/A4V}$ genotypes following axotomy. DNA was extracted from fixed neurons from three distinct platings in microfluidic devices and submitted for sanger sequencing. (**A**) Representative sequencing chromatogram of Exon 1 of SOD1 in SOD1$^{+/A4V}$ neurons. (**B**) Representative sequencing chromatogram of Exon 1 of SOD1 in SOD1$^{+/+}$ isogenic corrected control neurons.

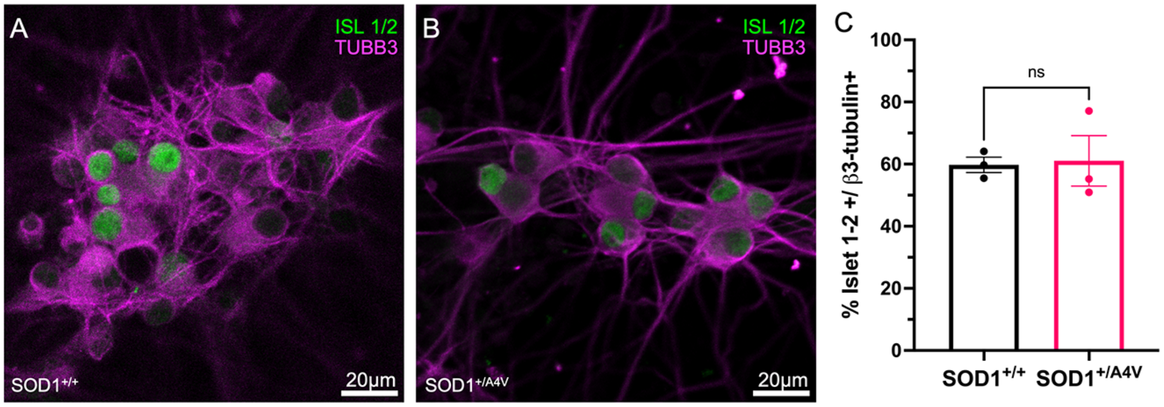

Supplementary figure 2: Efficiency of motor neuron differentiation for SOD1$^{+/+}$ and SOD1$^{+/A4V}$ is not different. (**A-B**) Representative images of (**A**) SOD1$^{+/+}$ and (**B**) SOD1$^{+/A4V}$ hiPSC-MNs immunostained for Islet 1/2 and β3-tubulin. Scale bars= 20μm. (**C**) Percentage of ISL1/2+ hiPSC-MNs (TUBB3+) for SOD1$^{+/+}$ (n=3 devices) and SOD1$^{+/A4V}$ (n=3 devices) following motor neuron differentiation for two independent platings from each line. Bars represent mean ± SEM. ns (not statistically significantly different) indicates p= 0.89.

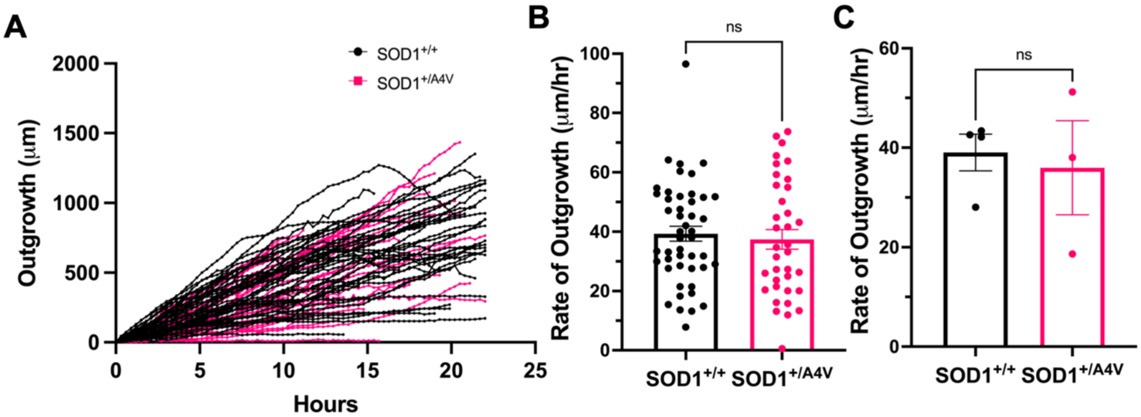

Supplementary figure 3: No significant difference in initial axonal outgrowth between $SOD1^{+/+}$ and $SOD1^{+/A4V}$. 24 hours after plating motor neurons in to the cell body compartment, live-cell imaging was used to track initial outgrowth of axons emerging from the channels into the axonal compartments to generate time-lapse video from which axons were tracked. (**A**) Outgrowth distance of individual axons following axotomy. Each line represents an individual axon. n= 3 sectors (33 axons) $SOD1^{+/A4V}$ and n= 4 sectors (47 axons) $SOD1^{+/+}$. (**B**) Average outgrowth speed. Each point represents the speed of an individual axon. (**C**) Average outgrowth speed. Each point represents the mean axon outgrowth speed from an individual sector (10-13 axons per sector).
